# CENsible: Interpretable Insights into Small-Molecule Binding with Context Explanation Networks

**DOI:** 10.1021/acs.jcim.4c00825

**Published:** 2024-06-07

**Authors:** Roshni Bhatt, David Ryan Koes, Jacob D. Durrant

**Affiliations:** † Department of Computational and Systems Biology, University of Pittsburgh, Pittsburgh, Pennsylvania 15260, United States; ‡ Department of Biological Sciences, University of Pittsburgh, Pittsburgh, Pennsylvania 15260, United States

## Abstract

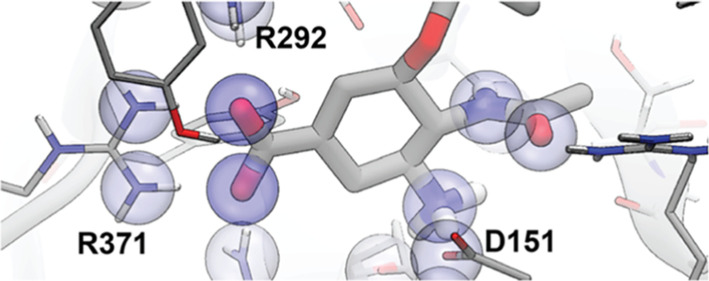

We present a novel and interpretable approach for assessing small-molecule binding using context explanation networks. Given the specific structure of a protein/ligand complex, our CENsible scoring function uses a deep convolutional neural network to predict the contributions of precalculated terms to the overall binding affinity. We show that CENsible can effectively distinguish active vs inactive compounds for many systems. Its primary benefit over related machine-learning scoring functions, however, is that it retains interpretability, allowing researchers to identify the contribution of each precalculated term to the final affinity prediction, with implications for subsequent lead optimization.

## Introduction

Structure-based computer-aided drug discovery (CADD) leverages computer algorithms to design and discover new drug candidates, with the twin goals of accelerating early stage drug design and reducing costs. Among CADD techniques, molecular docking is particularly popular. First, a docking program predicts how small-molecule ligands (e.g., candidate drugs) position themselves within a protein target’s binding pocket (the 3D geometry of binding, or “binding pose”). Second, a docking scoring function maps those binding geometries to scores correlating with efficacy (e.g., binding affinity). Researchers then submit the top-scoring compounds for experimental validation. Computer docking has a demonstrated history of success in both academic and industrial settings.^1,2^

Neural networks have emerged as a promising method for affinity prediction,^3−16^ but their predictions often lack interpretability, meaning that one cannot easily determine how they arrive at their conclusions. Poor interpretability complicates subsequent lead optimization. For example, neural network scoring functions do not typically indicate which atoms or functional groups contribute most to the overall binding affinity, insight that medicinal chemists could otherwise leverage to improve binding strength and specificity.

We present a novel, interpretable approach for assessing small-molecule binding using context explanation networks (CENs).^17^ Our CENsible scoring function uses deep neural networks to predict the contributions of precalculated physicochemical terms to a given binding affinity rather than directly predicting the affinity itself. In a sense, CENsible is similar to many traditional scoring functions, which predict binding affinities by summing the products of (1) calculated terms (e.g., hydrophobic and steric contributions to binding) and (2) fitted weights (coefficients). However, the CEN-derived weights are not the same for every protein/ligand complex. Instead, CENsible predicts the appropriate weights to apply to the calculated terms based on the specific structure of the protein/ligand complex. CENsible thus leverages the power of machine learning to predict affinity while retaining interpretability; one can easily identify the contribution of each precalculated term to the final prediction.

Users can download the CENsible source code free of charge without registration from https://durrantlab.com/censible/, under the terms of the GNU GPL license. The same site links to a helpful Google Colab.

## Results

Beyond predicting binding affinities, our CEN approach indicates the importance of different contributions to those affinities. For each example (e) of a protein/ligand complex cataloged in the PDBbind 2020 database,^18−22^ we used *smina* to precalculate a vector of scaled physicochemical terms, *t*_e_. We then trained a model with 8,269,024 fitted parameters (Figure S1) to learn from voxelized representations of the same complexes how to predict a vector of 144 weights, *w*_e_, specific to each protein/ligand complex, such that the linear combination of these two vectors, *w*_e_·*t*_e_, approximates the corresponding experimentally measured binding affinity.

### Scoring Function Accuracy

Our primary goal was to develop an interpretable machine-learning scoring function, but to have confidence in any such interpretation, the scoring function must also be reasonably effective at predicting binding affinity. To assess the accuracy and consistency of the CEN approach, we performed clustered 3-fold cross-validation using 15,895 examples of protein/ligand complexes present in the PDBbind 2020 database. We found that the CEN model effectively predicts binding affinities, with an average testing-set Pearson’s correlation coefficient of 0.5360 (Table 1). The accuracy of the model was fairly consistent regardless of the training/testing split used (i.e., the standard deviation was small), suggesting that (1) accuracy is more a product of learned affinity prediction than the specific data split and (2) the CEN models generalize to new protein classes. Similar analyses showed that the CEN approach performs well relative to several other scoring functions at assessing crystallographic poses (Tables S1–S3) and that our training protocol does not lead to severe overfitting (Figure S2).

**Table 1:** Pearson’s Coefficients Across Three Test Splits, Calculated via a Linear Fit between Known and Predicted Affinities

split 1 test	split 2 test	split 3 test	mean ± STD
0.5661	0.5543	0.4876	0.5360 ± 0.0423

Having shown that the three CENs have comparable accuracy regardless of the training/testing split chosen, we trained the final production model on all the PDBbind 2020 data (19,443 entries). We call this final production model the CENsible scoring function. The output vector of this scoring function includes 144 weights to be applied to 144 precalculated terms (Supporting Information 1). All subsequent analyses leveraged this production model.

The training data were split into thirds; for each fold, we trained a CEN model on two-thirds of the data and tested on the remaining third (top row). See Figure S3 for a graph showing how Pearson’s coefficients improved per epoch of training, as well as a scatter plot of experimentally measured vs predicted affinities.

### CENsible Tailors Scoring-Function Weights to Specific Targets

We hypothesized that if CENsible had truly learned to tailor scoring-function weights to binding-pocket properties, similar proteins should have similar predicted weight vectors (*w*_e_). To test this hypothesis, we identified SCOP families^23^ that were well represented among PDBbind 2020^18−22^ structures. Proteins belonging to the same SCOP family likely have similar binding pockets because, by definition, they must have at least 30% sequence identity, or some sequence identity in the context of very similar function.^23^

To assess whether proteins of the same family have similar CENsible-predicted *w*_e_ vectors, we projected the *w*_e_ vectors of 916 SCOP-classified PDBbind structures onto a two-dimensional space using the t-distributed stochastic neighbor embedding (t-SNE) method.^24^ We separately colored the structures belonging to well-represented SCOP families to highlight their distribution in this space.

**Figure 1:**
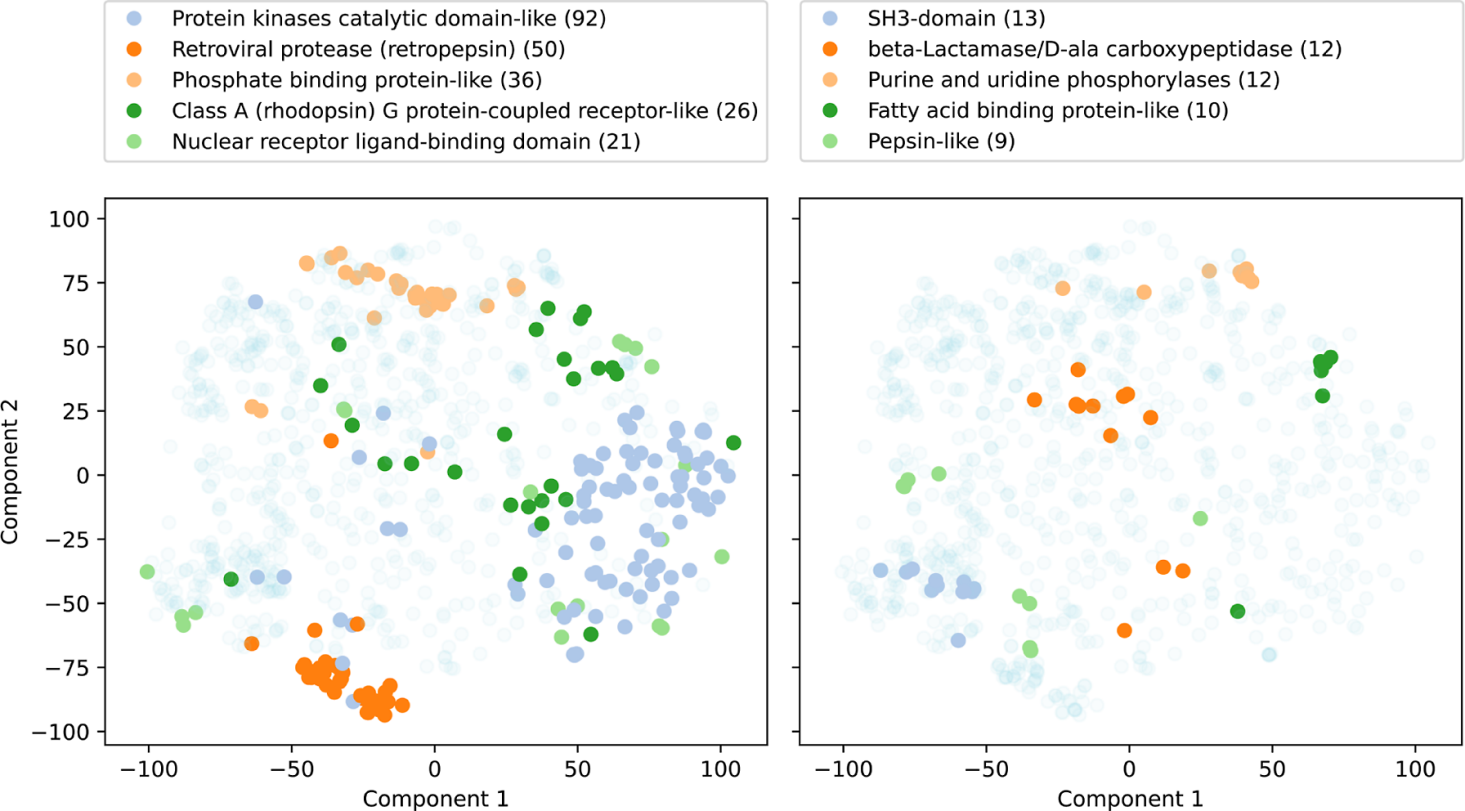
CENsible-predicted term weights (*w*_e_) projected onto a 2D t-SNE space, colored by the SCOP family label. The most-represented SCOP labels are colored with opaque markers; the remaining labels are colored with transparent markers. The top-five most represented SCOP families are shown in the left panel, and the next five are shown in the right panel. Family names are given above each panel, with the number of examples in parentheses.

Most of the SCOP families examined had predicted weights that were generally adjacent to each other in single “islands” in t-SNE space, including (1) protein kinases catalytic domain-like, (2) retroviral protease (retropepsin), (3) phosphate binding protein-like, (4) SH3-domain, (5) purine and uridine phosphorylases, and (6) fatty acid binding protein-like (Figure 1). This suggests that CENsible was fairly consistent in its weight predictions when assessing protein/ligand complexes from these families.

The predicted weights of four SCOP families were arguably more dispersed: (1) class A (rhodopsin) G protein-coupled receptor-like, (2) nuclear receptor ligand-binding domain, (3) beta-lactamase/d-ala carboxypeptidase, and (4) pepsin-like (Figure 1). These examples tended to congregate in multiple islands within the t-SNE space rather than one. Though it is possible that CENsible has simply not learned to predict consistent *w*_e_ vectors for these families, we note that it is also possible for similar vectors to be distant when projected onto t-SNE space in some cases.

These results suggest that CENsible generally produces customized and unique sets of weights for each protein/ligand complex based on the unique features learned from the voxelized representations of the complex. Similar protein/ligand complexes tend to have similar (albeit not identical) weights, and different protein/ligand complexes tend to have different weights.

### CENsible and Smina Capture Similar Physicochemical Contributions to Binding

To further assess whether CENsible had learned true principles of binding, we compared it to the more established *smina* scoring function, which does not leverage machine learning. Because CENsible and *smina* presumably model the same underlying physical reality, we hypothesized that they would predict affinity via similar principles (e.g., if *smina* judges that hydrophobic interactions contribute substantially to a given ligand’s affinity, CENsible should make the same general assessment).

Smina considers six physicochemical terms: a hydrogen-bond term, *non_dir_h_bond(g = −0.7,_b = 0,_c = 8)*; a hydrophobic term, *hydrophobic(g = 0.5,_b = 1.5,_c = 8)*; two steric terms, *gauss(o = 0,_w = 0.5,_c = 8)* and *gauss(o = 3,_w = 2,_c = 8)*; a repulsion term, *repulsion(o = 0,_c = 8)*; and a term that considers the number of rotatable ligand bonds, *num_tors_div*. It scales these terms by a single set of weights applicable to all protein/ligand complexes, derived by fitting to a diverse set of such complexes. Any term’s contribution to *smina*’s final affinity score is the product of the physicochemical term value and its associated weight.

Although CENsible incorporates a much larger set of 144 physicochemical terms, a given term’s contribution to the overall CENsible score is calculated similarly: the product of the physicochemical term value and its associated weight. However, CENsible calculates weights specific to each protein/ligand complex.

**Figure 2:**
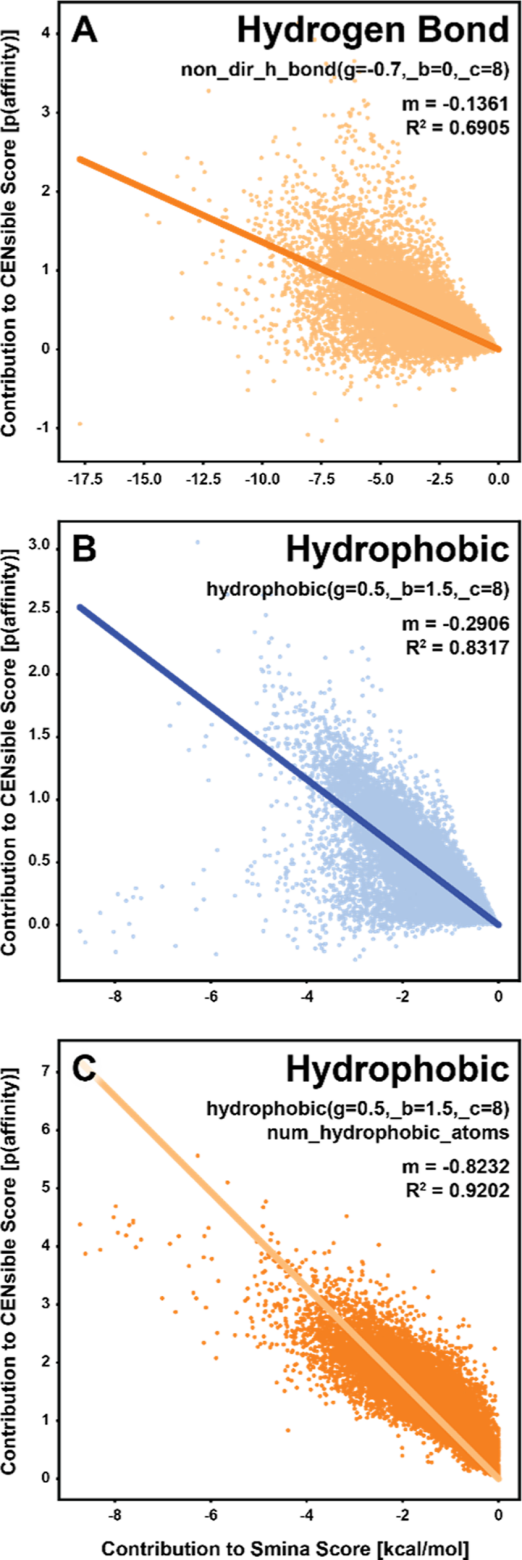
Correlations between *smina* and CENsible scoring contributions for shared physicochemical terms across ∼19,000 PDBBind protein–ligand complexes. (A) Hydrogen-bond term. (B) Hydrophobic term. (C) Hydrophobic term when the contributions of the *num_hydrophobic_atoms* term are additionally incorporated into the CENsible scores. All panels show linear regression lines that pass through the origin.

Five of *smina*’s terms are among the 144 that CENsible considers, so we focused on these for comparison. We first considered the hydrogen-bond term that *smina* and CENsible share, *non_dir_h_bond(g = −0.7,_b = 0,_c = 8)*. We calculated this term for the ∼19,000 protein/ligand complexes of the PDBBind database. For each complex, we multiplied the term by the appropriate *smina* weight (same value for all complexes) to determine its contribution to the final *smina* score. We similarly calculated the contributions to the respective CENsible scores by multiplying each physicochemical term by the complex-specific CENsible weight (different for each complex). The correlation (*R*^2^) between these contributions was 0.6905 (Figure 2A). Given that CENsible by design differs from *smina,* in that it tailors weights to each protein/ligand complex, we do not expect too strong a correlation between the two. Yet the fact that there is some correlation provides evidence that CENsible and *smina* capture similar underlying physical principles of binding.

We next considered the hydrophobic term that *smina* and CENsible share, *hydrophobic(g = 0.5,_b = 1.5,_c = 8)*. Here, too, the correlation between the contributions to the final *smina* and CENsible scores was high (*R*^2^ = 0.8317; Figure 2B), suggesting that both scoring functions capture the influence of this vital interaction, albeit not identically. CENsible also considers another term that likely correlates with hydrophobic interactions, *num_hydrophobic_atoms*. When we included this term in the hydrophobic contribution to the CENsible score, the correlation improved to 0.9202 (Figure 2C).

These results suggest that CENsible and *smina* have both learned similar principles of ligand binding. That said, CENsible can adapt those principles to the specific protein/ligand complex, perhaps explaining its improved accuracy over *smina* (Table S1). The Supporting Information describes similar comparative analyses applied to the steric and repulsion terms that *smina* and CENsible share (Figure S4).

### Virtual Screening Performance

To assess whether CENsible’s learned principles of ligand binding apply to docked poses, we applied the new scoring function to three benchmark virtual screens targeting *Homo sapiens* pancreatic glucokinase, influenza neuraminidase, and *Trypanosoma brucei* methionyl-tRNA synthetase, respectively. In the cases of *H. sapiens* pancreatic glucokinase and influenza neuraminidase, we used known actives and decoy compounds (presumed inactives) cataloged in the DUD-E database.^25^ In the case of *T. brucei* methionyl-tRNA synthetase, we used a compound library of known active and inactive compounds identified via PubChem.^26^

**Figure 3:**
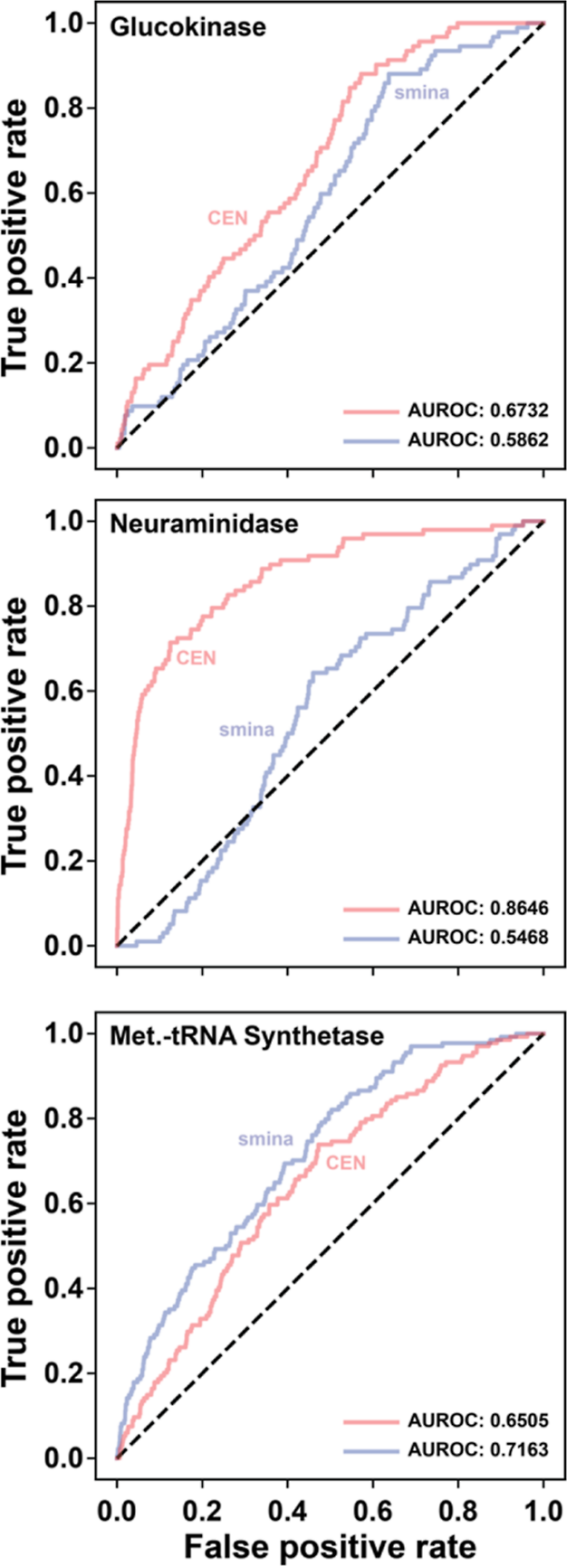
ROC curves associated with the *smina* (blue) and CENsible-rescored (red) virtual screens. The area under each curve is labeled as AUROC. The line of no-discrimination (dotted black line), corresponding to a purely random classifier, is shown for reference.

We used *smina*(27) to dock the active and inactive/decoy molecules associated with each protein into the respective active sites and rescored the *smina*-docked poses using our CENsible scoring function. We selected these screens because CENsible effectively prioritized known ligands over other compounds, suggesting that it had adequately learned binding principles for these proteins (Figure 3).

To evaluate the performance of each scoring function, we calculated the area under the receiver operating characteristic (ROC) curves (AUROC). We selected AUROC because it describes predictivity from the best-ranked compound to the worst and is insensitive to imbalanced data. We reason that if CENsible has learned general principles of ligand binding, it should be able to assess binding across a wide range of affinities.

Although our primary goal is interpretability, it is encouraging that, at least for some systems, CENsible can effectively rank docked poses, even though it was trained on crystallographic poses. The performance of the neuraminidase screens was particularly noteworthy (AUROC 0.8846).

### Neuraminidase: An Example of Interpretability

Because CENsible predicts weights to apply to precalculated terms rather than affinity directly, it offers valuable insights into molecular recognition that can guide subsequent lead optimization. For example, suppose a binding pocket contains a notable hydrophobic subpocket. Ligands with hydrophobic moieties positioned in that subpocket should have better scores than other compounds, and a scoring function that emphasizes hydrophobic contacts may better prioritize such molecules. In such a case, the CENsible-predicted weights on hydrophobic terms should ideally be higher, indicating that medicinal chemists should further consider hydrophobicity during lead optimization.

To provide a concrete illustration, we compared the weights predicted for the neuraminidase-docked compounds to those predicted for all PDBbind complexes in our training/testing sets. For reference, we first calculated the average predicted weights across the whole PDBbind data set, with standard deviations (Supporting Information 2). We then calculated the average weights across all neuraminidase-docked compounds. Finally, we calculated a z-score for each averaged neuraminidase weight with regard to the PDBbind reference.

**Figure 4:**
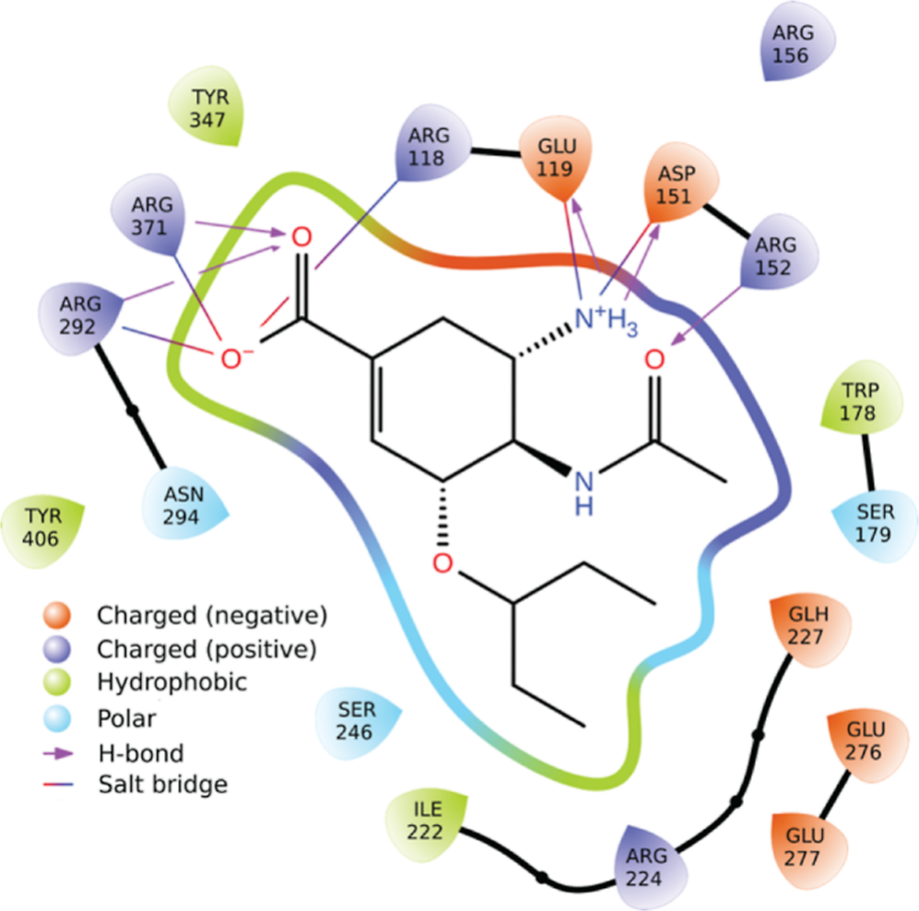
Interactions between influenza neuraminidase and the small-molecule ligand oseltamivir (PDB 2HU4), calculated using Schrödinger’s Ligand Interaction Diagram tool in Maestro.

Several neuraminidase weights with sizable z-scores suggest receptor-specific compound-optimization strategies. For example, many neuraminidase inhibitors (e.g., oseltamivir; Figure 4) have carboxylate groups that form electrostatic and hydrogen-bond interactions with ARG292, ARG371, and ARG118 (2HU4 numbering^28^). Indeed, CENsible tended to weigh electrostatics more heavily (Table 2). Many neuraminidase inhibitors also have moieties that bind in a hydrophobic subpocket lined in part by ILE222 (e.g., oseltamivir’s pentan-3-yloxy moiety; Figure 4). CENsible also tended to weigh the number of hydrophobic atoms more heavily when predicting neuraminidase inhibitors, perhaps to benefit compounds that could take advantage of this subpocket. This analysis suggests any ligand-optimization strategy should enhance (or at least preserve) these critical electrostatic and hydrophobic interactions.

**Table 2:** Select Precalculated Terms Whose Associated Averaged Predicted Neuraminidase Weights Differ Substantially from Those of the Entire PDBbind Set

precalculated term	NA weight^a^	PDBbind weight^b^	| *z*-score |
electrostatic(*i* = 1, ∧ = 100, *c* = 8)^c^	–7.52	–5.15 ± 1.72	1.38
electrostatic(*i* = 2, ∧ = 100, *c* = 8)^c^	–1.68	–1.27 ± 0.43	0.96
num_hydrophobic_atoms^d^	7.10	5.84 ± 1.42	0.88

a “NA weight” indicates the average CEN-predicted weight across all neuraminidase-screen evaluations.

b “PDBbind weight” indicates the average weight (±standard deviation) across all PDBbind complexes used for training and testing.

c The two electrostatic terms are calculated by considering all pairs of atoms within 8 Å of each other. The partial charges of each atom are multiplied and then divided by either the distance (*i* = 1) or the distance squared (*i* = 2), with each pair’s value capped at 100. The final value is calculated by summing over all atom pairs. Negative electrostatic values are more energetically favorable, so more negative electrostatic weights translate to larger (better) CENsible scores.

d The *num_hydrophobic_atoms* precalculated term is a simple count of the hydrophobic atoms present in the ligand. It is always positive, so more positive weights translate to better CENsible scores.

### Visualizing the Per-Atom Contributions of the Gaussian Steric Terms

Of the 144 precalculated terms that CENsible considers, 123 are pairwise steric (*atom_type_gaussian*) terms. In brief, for each pair of receptor/ligand atoms within 8 Å of each other, one calculates the (1) interatomic distance, *d*, and (2) the “optimal distance,” *d*_0_ (i.e., the summed radii of the two atoms). The final value, *g*, is assigned to that pair according to the formula
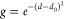


For each combination of receptor/ligand atom types, the final *atom_type_gaussian* term is calculated by summing the associated atom-pair *g* values.

Because these terms are among those directly attributable to specific pairs of receptor/ligand atoms, it is possible to visualize the impact of each atom on each of CENsible’s *atom_type_gaussian*-associated contributions. We calculate the *g* value for each pair, scale that value by the same factor CENsible applies to the corresponding precalculated term, and multiply by the associated CENsible-predicted weight. Finally, we assign half of this *g* value to the receptor atom and half to the ligand atom. Where a single atom contributes to multiple atom-type pairs, the associated values are summed.

**Figure 5:**
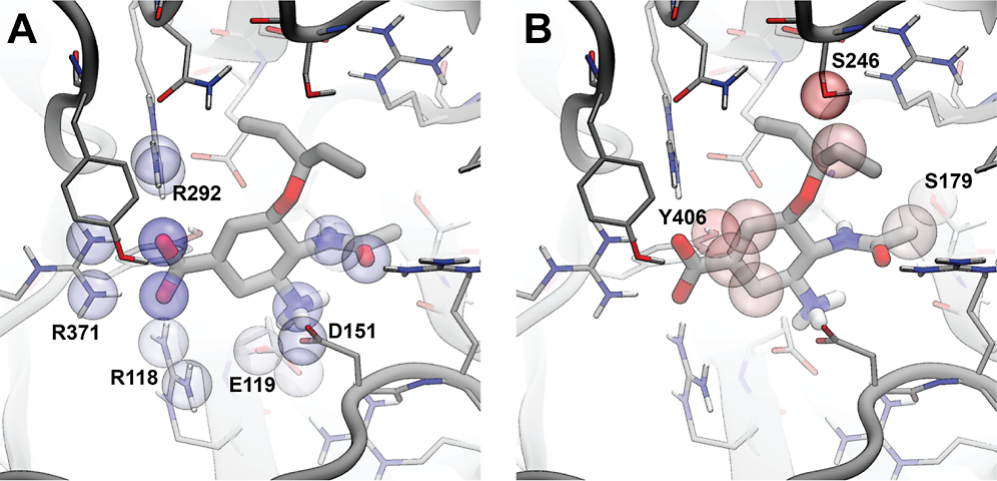
Illustration of the interactions between neuraminidase and oseltamivir. Panel (A) shows the contributions of beneficial protein/ligand contacts that participate in the *NitrogenXSDonor-OxygenXSAcceptor atom_type_gaussian* term. Panel (B) shows the contributions of detrimental contacts associated with the *AliphaticCarbonXSHydrophobe*-*OxygenXSDonorAcceptor* term. To simplify the presentation, we highlight only receptor atoms that come within 4 Å of the ligand. We further highlight only those receptor/ligand atoms that contribute more than +0.025 or less than −0.025 to the final CENsible score. Colors range from red to white to blue over the numerical range −0.15 to 0.0 to +0.15. Values outside that range were set to the nearest range boundary.

We applied this analysis to the same neuraminidase example above. The most beneficial contribution was associated with the *NitrogenXSDonor-OxygenXSAcceptor atom_type_gaussian* term, which contributed +1.05 to CENsible’s final score of 7.45. As shown in Figure 5A, this term captures the same electrostatic interactions seen above (i.e., interactions between the oseltamivir carboxylate group and ARG292, ARG371, and ARG118, as well as interactions between the oseltamivir amine and ASP151 and GLU119), further boosting the influence of electrostatics on the final score (see Table 2).

The *AliphaticCarbonXSHydrophobe-OxygenXSDonorAcceptor atom_type_gaussian* term had the most detrimental impact, decreasing CENsible’s final score by −0.507. Biochemically, it makes sense that this term would have a negative effect, as the juxtaposition of hydrophobic carbon atoms and hydrophilic hydrogen-bond donor/acceptor groups is typically unfavorable. Notably, the SER246 hydroxyl group is near oseltamivir’s pentan-3-yloxy moiety, and the TYR406 hydroxyl group is near its central carbon ring (Figure 5B). Additionally, SER179 is near an oseltamivir methyl group. These findings suggest that modifying oseltamivir to better form hydrogen bonds with these residues could enhance affinity.

## Discussion

We present a novel approach for assessing protein/ligand binding using a CEN. Like other machine-learning approaches, our method has learned principles of ligand binding. However, our model is far more interpretable and so can help guide subsequent lead optimization. To our knowledge, this study describes the first time CENs have been applied to protein/ligand scoring.

While CENsible is impressively accurate at predicting binding affinities for some systems (e.g., neuraminidase, Figure 3), its primary value lies in the interpretability it provides via its predicted weights (Table 2 and Figure 5). Predicting which weights to apply to the precalculated physicochemical terms is not merely a means to an accurate-affinity-prediction end; rather, the weights themselves—and the interpretability they enable—are the end goal. From our perspective, reasonably accurate affinity prediction is important only in that it gives us confidence in those interpretable weights.

Users who wish to use CENsible primarily for affinity prediction rather than interpretability should be wary of several factors that can impact those predictions. First, CENsible affinity predictions depend on the accuracy of the underlying (docked) poses (Figure S5); CENsible is less likely to accurately predict affinities if given incorrect poses. Second, even if the underlying poses are accurate, CENsible is not suited to every system (Figure S6). As others have noted, the same is true of any docking scoring function.^15,18,29−31^ For those systems where CENsible cannot reasonably predict affinity, we do not recommend further using it to gain interpretable insights into binding.

Third, even when CENsible’s accuracy against a given target is sufficient to inspire confidence in its predicted weights, it may not be the best scoring function for the specific task of affinity prediction. For example, rescoring the *smina*-docked poses from the glucokinase and methionyl-tRNA synthetase screens with the *gnina* default scoring function gave ROCAUC values of 0.8120 and 0.7225, higher than those obtained when rescoring with CENsible (0.6732 and 0.6505). In contrast, CENsible performed somewhat better than *gnina* default on the neuraminidase screen (0.8646 vs 0.8126). Per the top-compound enrichment-factor metric (i.e., the extent to which the top-ranking compounds are enriched with true binders), even *smina* outperforms CENsible in some cases, though in others, CENsible is the clear winner (e.g., the glucokinase and neuraminidase screens; Figure S7). Regardless, CENsible’s primary advantage lies in its ability to provide interpretable output tailored to a specific protein/ligand complex.

We also recommend some caution when visualizing the per-atom contributions of the Gaussian steric terms (Figure 5). While this visualization can provide interpretable information to guide subsequent lead optimization, it may omit some relevant contributions to binding because CENsible relies in part on overlapping precalculated terms. For example, when scoring potential neuraminidase ligands, CENsible predicted a relatively large weight for the *num_hydrophobic_atoms* term (Table 2), a term that depends only on ligand atoms. However, several *atom_type_gaussian* precalculated terms also capture hydrophobicity (e.g., the *AliphaticCarbonXSHydrophobe*-*AliphaticCarbonXSHydrophobe* term). Other types of interactions are similarly associated with overlapping, though not identical, terms. Using the *atom_type_gaussian* terms alone to assess the importance of such interactions is thus ill-advised, given that much of their contribution could reside in other terms. Indeed, the summed contribution of all *atom_type_gaussian* terms to a final CENsible score is often only a fraction of the total (e.g., 1.06/7.45 for the neuraminidase/oseltamivir complex).

These limitations aside, we are hopeful that CENsible will be a useful tool for the CADD community. We release it under the GNU GPL license. The software is available for download from https://durrantlab.com/censible/ free of charge, without registration, and is designed to run on Linux-like operating systems. It requires Python3 (tested on version 3.9.16), the *obabel* (Open Babel) executable to process user-provided protein and small-molecule structures (e.g., to remove water molecules, protonate at pH 7, etc.), and the *smina* executable to calculate precalculated terms for each protein/ligand complex. The git repository includes a helpful README.md file with installation and usage instructions. To encourage broad adoption, we also provide a Google Colab, accessible via https://durrantlab.com/censible/ (Supporting Information 3).

## Online Methods

### Data Used for Training and Testing

We downloaded data for training and testing from the PDBbind 2020 database,^18−22^ which includes 19,443 crystal structures of protein/ligand complexes taken from the Protein Data Bank with associated experimentally measured binding affinities. We applied several criteria to process this data set. First, we retained all entries with precisely and approximately defined binding affinities (denoted with “=” and “∼”, respectively). Second, we removed entries with binding affinities measured only as being weaker than a given value (denoted with “>”), judging these to be ambiguously defined. Third, we similarly discarded entries measured only as being stronger than a given value (denoted with “<”) if the associated value was greater than 1 μM; otherwise, these entries were retained. After applying these filters, 19,193 entries remained.

We used a clustered 3-fold cross-validation scheme to split this data into training and testing sets. We downloaded the Protein Data Bank’s weekly clustering of protein sequences (30% sequence identity)^32−34^ on October 3, 2023. To avoid ambiguity, we removed any multichain PDBbind structures belonging to two or more clusters, such that 15,895 PDBbind entries remained. We divided these data into three independent portions, ensuring that entries belonging to the same cluster were always assigned to the same portion. Finally, we trained three different models on two portions, withholding the third as an independent testing set in each case.

### Data Preparation for Training

We standardized all PDBbind protein/ligand structures before training. We removed water molecules and used Open Babel^35^ to add protein and ligand hydrogen atoms appropriate for pH 7. We then voxelized each protein/ligand complex using the *libmolgrid*(36) python package with default parameters (e.g., resolution of 0.5 Å, 48 × 48 × 48 grid points, 28 atom types; Supporting Information 4). Voxelization converts a continuous 3D space (e.g., the Cartesian coordinates of atomic positions) into a discrete 3D grid well suited as input data for machine-learning models such as convolutional neural networks.

For each PDBbind entry, we also precalculated 348 physicochemical terms using the *smina* executable,^27^ based on the structure of each protein/ligand complex (Supporting Information 1). These included terms used in the AutoDock 4^37^ and AutoDock Vina^38^ scoring functions, terms specific to the *smina* scoring function,^27^ counts and whole-ligand features (e.g., ligand length and number of ligand heavy atoms), and a set of 325 steric terms that describe interactions between atoms of specific types (e.g., AliphaticCarbonXSNonHydrophobe vs OxygenXSAcceptor; *smina* keyword *atom_type_gaussian*). Many of these 325 steric terms were frequently zero; we discarded any that were nonzero in less than 1% of the examples used for training and testing. See Supporting Information 1 for a complete list of the terms retained in the final scoring function.

The precalculated terms had different units and ranges, which could introduce bias into our model. To ensure that all terms were of comparable magnitude, we normalized the data by scaling all terms (i.e., those associated with both training and testing sets)
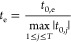
where *t*_e_ is the scaled term associated with a given protein/ligand example, e; *t*_0,e_ is the original unscaled term; and *T* is the total number of examples across the training and testing sets. This approach ensured that all terms fell within the range −1 to 1 without changing the sign of any term, which is sometimes physically meaningful.

### Model Training

The CEN scoring functions in the present work use the same deep convolutional neural network architecture as *gnina*’s default2018 scoring function,^14,39,40^ except instead of predicting a single value (binding affinity), they predict a vector of weights (Figure S1). For discussion’s sake, we use *w*_e_ to refer to the output weight vector associated with a given protein/ligand example, e. These weights serve as coefficients on the scaled precalculated terms (*t*_e_), such that *w*_e_·*t*_e_ approximates the corresponding experimentally measured binding affinity (i.e., pIC_50_, p*K*_d_, and p*K*_*i*_ values derived from the PDBbind 2020 database^18−22^).

We trained the CENs for 250 epochs on an NVIDIA GeForce RTX 3090 GPU using the stochastic gradient descent optimizer (learning rate: 0.01; weight decay: 0.0001; momentum: 0.9; see Figure S3) and the StepLR scheduler (step size: 80; gamma: 0.1). Each time a voxel grid was used for training, it was translated by at most 2 Å and randomly rotated. The loss was calculated using the smooth L1 loss criterion,^41^ which compared experimental and predicted binding affinities (*w*_e_·*t*_e_). The loss was calculated per batch (batch size: 25).

### Model Assessment

We used two methods to assess the predictions of the CEN models. First, we used Pearson’s correlation coefficients to quantitatively assess the accuracy of our model predictions on a withheld test set by comparing predicted and experimentally measured binding affinities.

Second, we used the t-distributed stochastic neighbor embedding (t-SNE) method,^24^ implemented in the scikit-learn Python package,^42^ to project the CEN-predicted weights onto a two-dimensional space for visualization. *K*-means clustering verified that similar weight vectors generally map to adjacent regions of this two-dimensional space (Figures S8 and S9). We then identified 916 entries in the PDBbind 2020 database^22^ (used to train CENsible) that also had entries in the SCOP database (release 2022-06-29) with only one unambiguous protein-family assignment. Finally, we identified the SCOP families that were most frequently represented among the labeled PDBbind structures and projected them onto the same t-SNE space with per-family coloring.

### Virtual Screen: *Homo sapiens* Pancreatic Glucokinase

To confirm that CENsible has learned the principles of binding required to separate true ligands from decoys, we performed a benchmark virtual screen targeting *H. sapiens* pancreatic glucokinase, leveraging the files associated with the *HXK4* entry in the DUD-E database.^25^ The receptor file associated with this entry was derived from the PDB 3F9M structure.^43^ The entry also includes 92 known active molecules, as well as 4696 decoys (active-to-decoy ratio of roughly 1:51). The DUD-E receptor and compounds already have assigned protonation states and so required no further processing.

We used *smina*(27) to dock each compound into a box centered on the glucokinase active site. We used *smina*’s “autobox_ligand” parameter to determine the appropriate box dimensions from the DUD-E-provided crystallographic ligand (ligand ID: MRK) and *smina*’s default parameters otherwise. To assess accuracy (e.g., AUROC and EF %), we considered only the top-scoring *smina* pose per ligand, regardless of ionization, tautomerization, etc. We rescored that *smina* pose with our CENsible scoring function.

### Virtual Screen: Influenza Neuraminidase

We also performed a virtual screen targeting influenza neuraminidase, leveraging the files associated with the *NRAM* entry in the DUD-E database.^25^ The *NRAM* receptor file was derived from the PDB 1B9V structure.^44^ The entry includes 98 known active molecules, as well as 6200 decoys (active-to-decoy ratio of roughly 1:63). The DUD-E receptor and compounds already have assigned protonation states, and so required no further processing.

We used *smina*(27) to dock each compound into a 20 Å × 20 Å × 20 Å box centered on the neuraminidase active site. We used *smina*’s default parameters otherwise. We again assessed accuracy using AUROC and EF % as above and similarly rescored the *smina* poses with our CENsible scoring function.

### Virtual Screen: *Trypanosoma brucei* Methionyl-tRNA Synthetase

We also performed a virtual screen targeting *T. brucei* methionyl-tRNA synthetase. To prepare the *T. brucei* methionyl-tRNA synthetase structure for docking, we downloaded entry 4EG4^45^ from the Protein Data Bank.^33^ We processed the file using the Protein Preparation Workflow available in Schrödinger Maestro 13.5.128 (default parameters). We then removed water and small-molecule-ligand residues and converted *S*-(dimethylarsenic)cysteine (CAS) residues to cysteine residues.

To prepare small-molecule compounds for the virtual screen, we downloaded the SMILES strings associated with PubChem^26^ bioassay 624268 (primary screen) and bioassay 651971 (confirmatory screen).^46^ We selected the 134 most active compounds from the confirmatory screen (IC_50_ < 5 μM). We also randomly selected 5360 inactive compounds from the primary screen, such that the active-to-inactive ratio was 1:40. We processed the SMILES strings using Schrödinger’s LigPrep utility to generate 3D structures with enumerated ionization, tautomerization, and chiral states (default parameters, except we generated only at most two stereoisomers per input molecule).

We used *smina*(27) to dock each compound into a 20 Å × 20 Å × 20 Å box centered on the methionyl-tRNA synthetase active site, using *smina*’s default parameters. We again assessed accuracy using AUROC and EF % as above and similarly rescored the *smina* poses with our CENsible scoring function.

### Assistive Writing Technologies

We used assistive writing technologies such as Grammarly and OpenAI’s ChatGPT during manuscript preparation. These supplementary tools acted as editors, not as drivers of content creation. The listed authors thoroughly reviewed, revised, and selectively implemented the suggested edits to ensure accuracy, consistency, and clarity. The responsibility for the paper’s content and quality remains with the authors alone.

## Data Availability

Source data are included in the Supporting Information. The PDBbind 2020 database used for training and testing is available for download from http://www.pdbbind.org.cn/. The computer code associated with this study is available through https://durrantlab.com/censible/. The same page links to a Google Colab that implements the CENsible approach. A copy of the Google Colab Python notebook is included as Supporting Information 3.

## Supporting Information Available

Additional information regarding training, weight-vector clustering, the CENsible-rescored virtual screens, precalculated terms, CENsible-predicted weights of all PDBbind protein/ligand complexes used for training and testing, a Google Colab Python notebook for rescoring protein/ligand complexes using CENsible, and the receptor and ligand atom types used to generate the voxel grids (PDF)
Precalculated term names and whether the term names are included in the production model (XLSX)
Grid atom types (PDF)
PDBbind predicted weights (XLSX)
CENsible (ZIP)
SMILES (TXT)
