# Supplementary material for: CENsible: Interpretable Insights into Small-Molecule Binding with Context Explanation Networks
Source: J Chem Inf Model. 2024 Jun 7;64(12):4651–60. doi: 10.1021/acs.jcim.4c00825 (PMC11200255; doi:10.1021/acs.jcim.4c00825)
Supplement: Supplementary file 1 — ci4c00825_si_001.pdf [file ci4c00825_si_001.pdf]

Supporting Information

CENsible: Interpretable Insights into Small-Molecule Binding with Context Explanation Networks

Roshni Bhatt[1,2], David Ryan Koes[1], Jacob D. Durrant[2 *]

[1] University of Pittsburgh, Department of Computational and Systems Biology, Pittsburgh, PA, US 15260

[2] University of Pittsburgh, Department of Biological Sciences, Pittsburgh, PA, US 15260

[*] Correspondence: Jacob Durrant, Department of Biological Sciences, University of Pittsburgh, Pittsburgh, PA 15260. Email: durrantj@pitt.edu

CENsible architecture

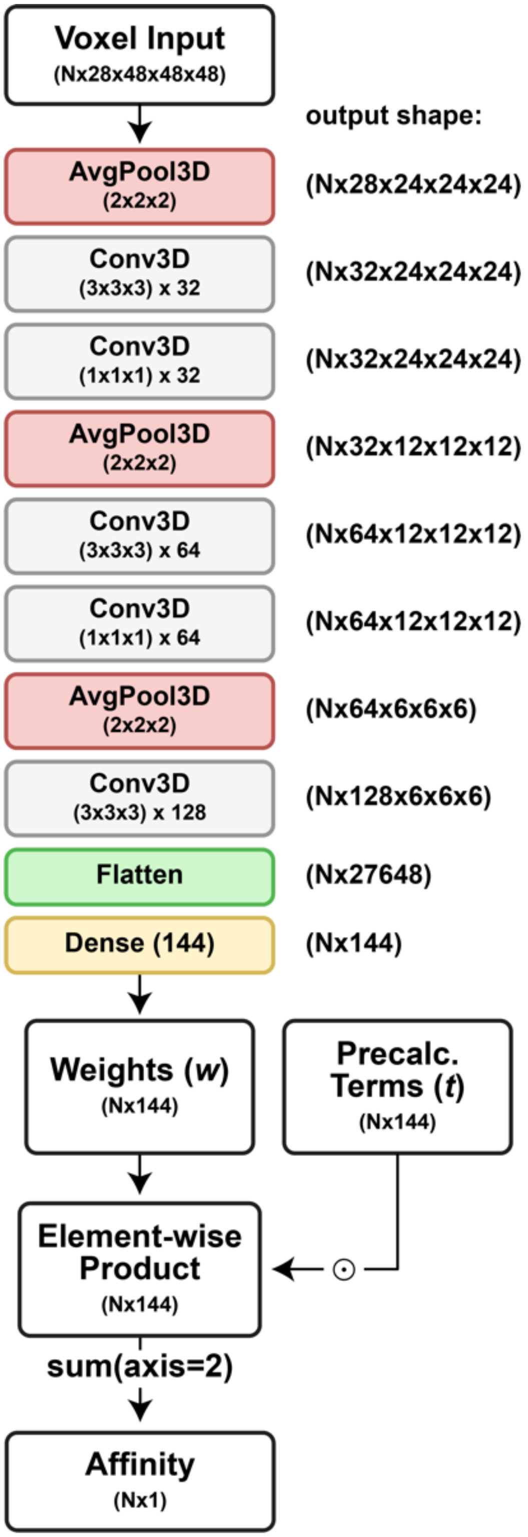

Figure S1. CENsible has the same architecture as *gnina*'s *default2018* model, except the final layer outputs weight vectors (coefficients) rather than affinities. Each Conv3D layer is followed by a rectified linear unit (RELU), not shown. *N* is batch size. In total, there are 8,269,024 fitted parameters. To calculate the loss per batch, the model determines per-batch affinity predictions by taking the row-wise dot products of these weights and the precalculated terms.

Evaluating Overfitting

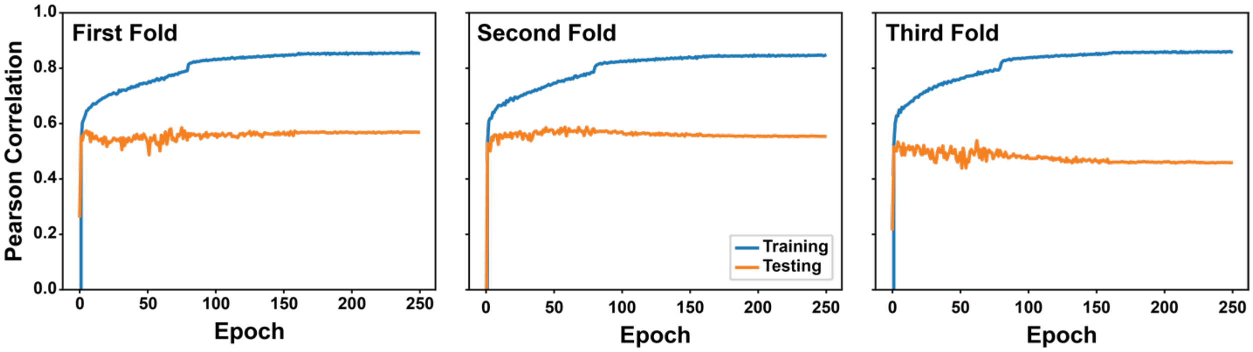

Figure S2. Evaluation of model overfitting. Pearson correlation coefficients for training (blue) and testing (orange) sets are plotted across epochs for three distinct folds. The performance on the testing sets does not degrade over time, and all three models converge to similar coefficients, indicating consistent generalization across different data subsets and suggesting that overfitting is not severe.

To assess whether our training protocol is given to overfitting, we retrained the same three-fold cross-validation models described in Table 1, this time calculating the Pearson correlation coefficients for all training-set and testing-set predictions per epoch (Figure S2). As expected, each model is better at predicting the affinities of examples in its training set. This difference is typical because models generally perform better on the data used for training, but it is especially true in this case, given our efforts to ensure that training and testing sets were independent in terms of protein-sequence similarity. Most importantly, the testing set's performance does not substantially degrade over time, suggesting that overfitting is not severe.

The three models also converge to roughly the same Pearson correlation coefficients, even though they are evaluated on different testing sets. This consistency suggests that the models generalize well, indicating that severe overfitting to the training data is unlikely.

Training Evaluation

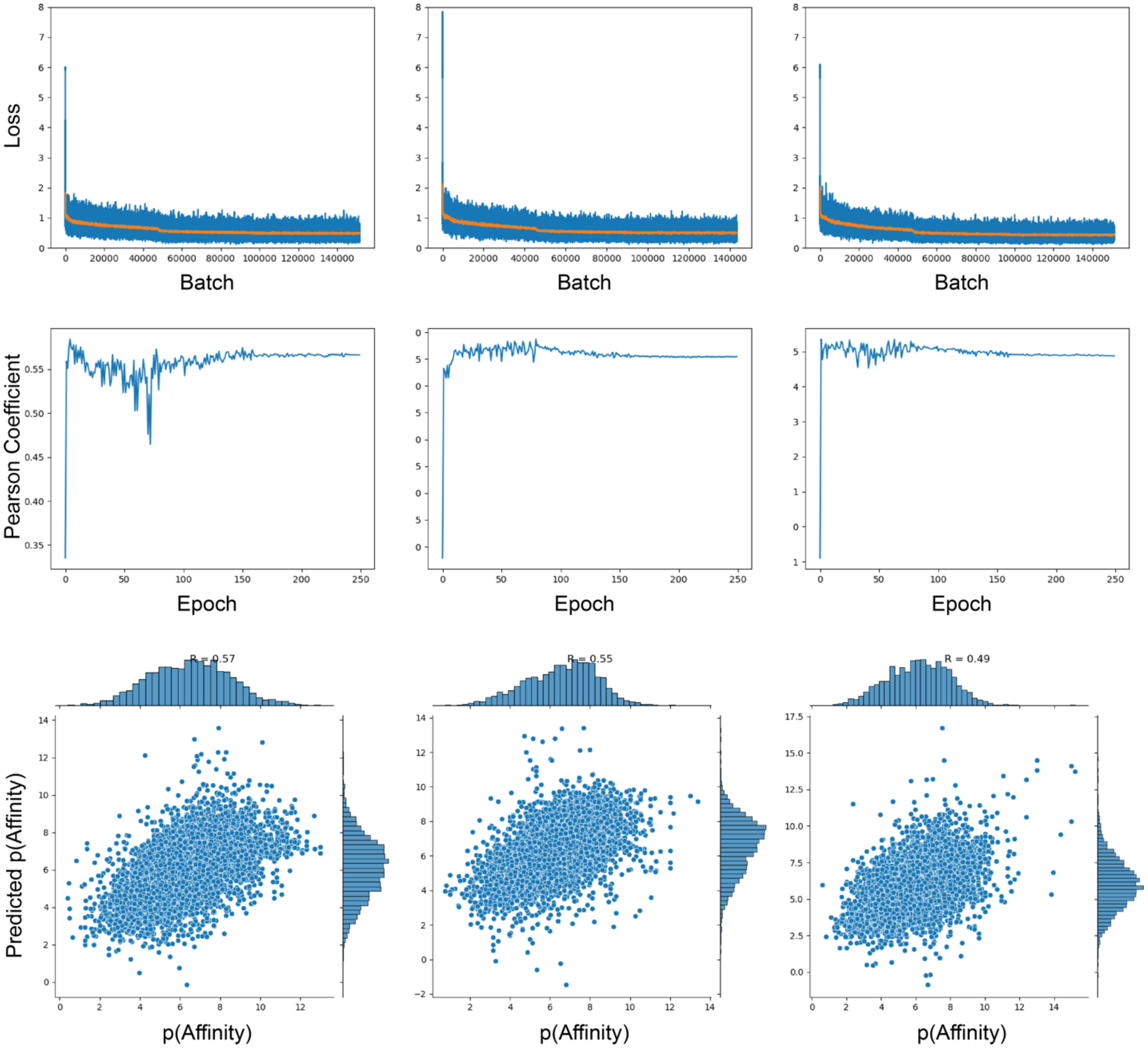

Figure S3. Training evaluations for the first (left), second (middle), and third (right) folds when using clustered cross-validation. These analyses were performed using the same models described in Table 1. Top row: the loss per batch. The orange line represents a moving average of window size 100. Middle row: the Pearson coefficient on the withheld test set for each fold, per epoch of training. Bottom row: a scatter plot of experimental vs. CENsible-predicted affinities (negative logarithm transforms).

CENsible and Smina Capture Similar Physicochemical Contributions to Binding

Following the approach detailed in the main manuscript, we investigated the correlation between the steric- and repulsion-term contributions to the *smina* and CENsible scores.

To assess steric contributions to binding, we considered the *gauss(o=0,_w=0.5,_c=8)* and *gauss(o=3,_w=2,_c=8)* terms, adding these weighted terms together to capture their combined contributions to the final scores. The steric contributions associated with the two scoring functions are again highly correlated (Figure S4A), suggesting that both scoring functions generally capture the same binding principles.

To assess the repulsion-term contributions to binding, we considered the *repulsion(o=0,_c=8)* term, which *smina* and CENsible also have in common. The analysis again revealed a strong correlation (Figure S4B); however, we found that CENsible systematically scales down the weight on the repulsion term, making its contribution to the overall score far smaller than in *smina*. The repulsion-term contribution to a few of the PDBBind crystal structures is very large; we suspect that CENsible has reduced the influence of this term to overcome this apparent artifact.

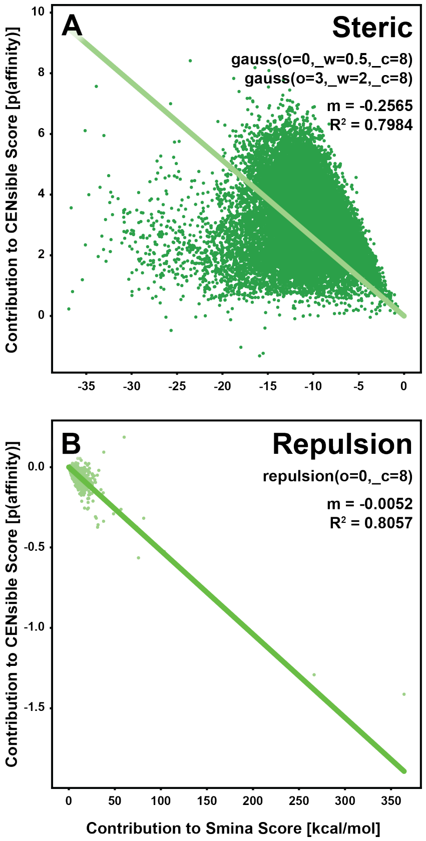

Figure S4. Correlations between *smina* and CENsible scoring contributions for shared physicochemical terms across ~19,000 PDBBind protein-ligand complexes. A) Combined steric terms. B) Repulsion term. All panels show linear regression lines that pass through the origin.

CENsible Scores Depend on Accurate Poses

To provide evidence that CENsible scores depend on the accuracy of the underlying ligand poses, we considered all the known-active compounds from the three virtual screens presented in the main text: glucokinase, neuraminidase, and methionyl-tRNA synthetase. We rescored these compounds' best-scoring and worst-scoring *smina* poses with CENsible. We hypothesize that CENsible leverages information about the protein/ligand complex to make its assessments, so the CENsible scores associated with the worst-scoring (likely incorrect) poses should be less than those associated with best-scoring (more likely to be correct) poses.

As shown in Figure S5, this is indeed the case. We used SciPy's[1] *stats.mannwhitneyu* function to perform three two-sided Mann-Whitney U tests[2] to assess whether the differences between rescored best/worst *smina* poses are statistically significant. We selected this approach because Shapiro–Wilk tests[3] suggested that some of the score distributions shown in Figure S5 are not normally distributed, complicating the use of a straightforward Student's t-test[4]. Per this assessment, the median values are statistically different in all three cases. Further, in all three cases, the median score of the rescored best-scoring *smina* pose was higher (better) than that of the rescored worst-scoring *smina* pose.

These results suggest that CENsible scores improve when reassessing accurate poses, further implying that CENsible's assessment does depend on the structure of the protein-ligand complex.

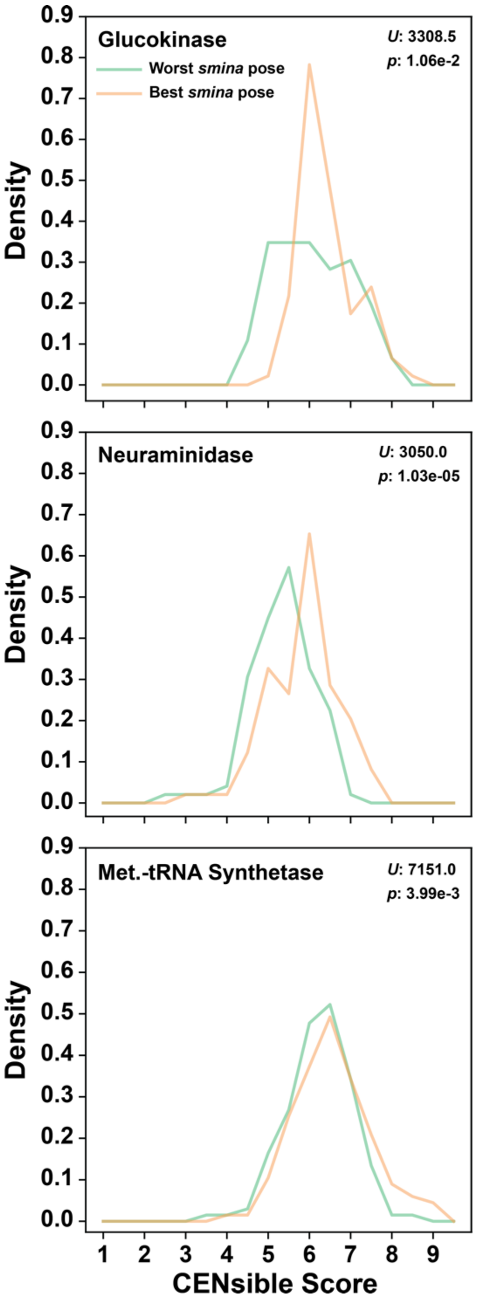

Figure S5. Probability distributions of CENsible-rescored *smina* poses. Only known active compounds from the three virtual screens are included. Bins are spaced 0.5 units, though only every other bin is marked on the X axis. The score distributions associated with the worst and best *smina*-docked poses are shown in green and orange, respectively. U statistics and p values are given in the upper-right corner of each panel.

CENsible is not Well Suited to All Systems

Though CENsible generally performs better than *smina* (Pearson's coefficients on testing sets; Table S1), it is not well suited to all systems, as is generally the case with all scoring functions[5-9]. A benchmark virtual screen targeting HIV integrase illustrates this variability. The protein and compounds for this screen were again sourced from the DUD-E dataset (id *HIVINT*). The foundational *smina* screen was notably predictive (AUROC: 0.7864; Figure S6). However, when the *smina* poses were reassessed using CENsible, there was a marked decline in the AUROC (0.4949; Figure S6). Similarly, rescoring the poses with the default *gnina* scoring function[10,11] also reduced the AUROC considerably (0.5237, data not shown).

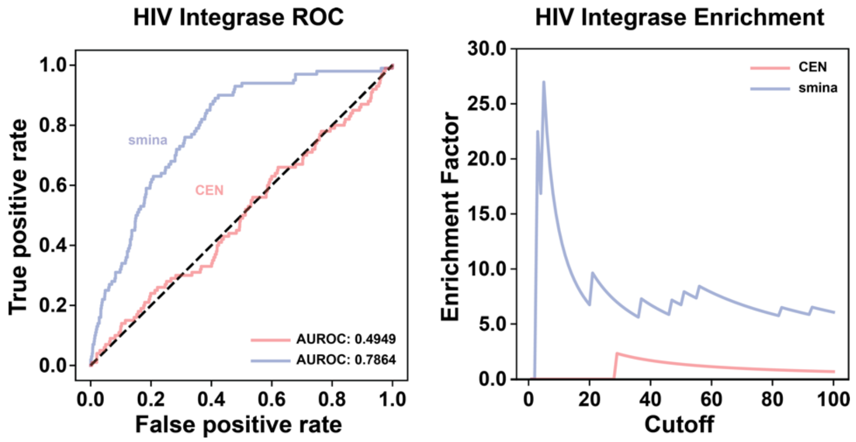

Figure S6. A virtual screen targeting HIV integrase illustrates that CENsible, like all scoring functions, is not well suited to every receptor. On the left, the ROC curves associated with the *smina* (blue) and CENsible-rescored (red) virtual screens. The area under each curve is labeled as AUROC. The line of no-discrimination (dotted black line), corresponding to a purely random classifier, is shown for reference. On the right, the top-compound enrichment factors (i.e., the extent to which the top-ranking compounds are enriched with true binders).

Virtual Screen Enrichment Factors

In the main text, we use three benchmark virtual screens to assess whether CENsible's learned principles of ligand binding apply to docked poses. We reason that if CENsible has truly learned general principles of ligand binding, it should be able to assess binding across a wide range of affinities. We thus used the AUROC metric to assess these screens (Figure 3) because it captures predictivity from the best-ranked compound to the worst.

Enrichment factors (EFs) are also useful for assessing virtual-screen predictivity. These factors capture the extent to which the top-ranked compounds are enriched with true binders. The EF metric is useful when the goal is not to interpretably assess ligand binding but rather to identify novel ligands. Though EFs are not ideally suited for our assessment, we include them here for completeness.

By the EF metric, CENsible performs well relative to *smina* on the glucokinase and (especially) the neuraminidase screens, but *smina* has better enrichment in the *T. brucei* methionyl-tRNA synthetase screen (Figure S7). Regardless, CENsible's primary utility lies in its ability to provide interpretable output tailored to a specific protein receptor.

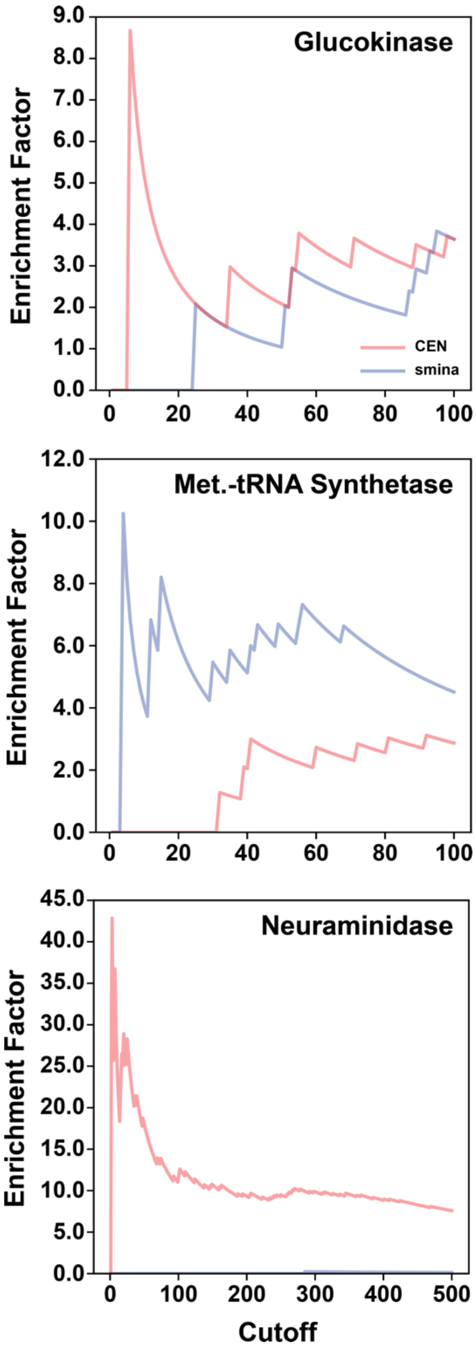

Figure S7. Top-compound enrichment factors (i.e., the extent to which the top-ranking compounds are enriched with true binders).

Comparison with Other Scoring Functions at Evaluating Crystallographic Poses

Our primary goal was to develop an *interpretable* machine-learning scoring function, but we were also curious how well our approach could score crystallographic poses compared to other related functions (e.g., *smina*[12] and the *dense*, *gnina2018*, *gnina*, *crossdock_default2018*, *redock_default2018*, and *default2017* neural-network scoring functions available via the *gnina* software package[10,11]).

We could not use the *clustered* cross-validation approach described in the main text to create independent training and testing sets for these other scoring functions because we do not know which proteins were included in their training sets. To ensure a fair comparison, we therefore trained three new CEN models using *unclustered* threefold cross validation. Specifically, we randomly split the PDBbind data into three folds without regard for sequence similarity. We trained three new CEN models on different combinations of two portions, withholding the third as a testing set in each case.

Table S1 shows that the three CEN scoring functions performed better than *smina*. This comparison is critical because the CEN scoring functions incorporate some *smina* terms. The difference is that *smina* uses a single set of weights for all protein/ligand complexes, but the CENs essentially customize the weights based on the structure of the protein/ligand complex.

We also evaluated the same poses present in each cross-validation testing set using *gnina* neural-network-based scoring functions[10,11]. Table S1 shows that the CENs are substantially better at predicting the binding affinities of crystallographic protein/ligand complexes than even the best of the *gnina* scoring functions. Given these results, it is tempting to conclude that the CEN scoring functions are better suited for prospective virtual screens than *gnina* functions. We note, however, that the best performing *gnina* function (*gnina default*) is an ensemble of five models, some of which were trained on docked rather than crystallographic data. The *gnina default* function may therefore be more predictive in the context of a virtual screen[10,11]. Regardless, the strength of the CEN approach is that it is far more interpretable.

Table S1. Pearson's coefficients across three test splits, calculated via a linear fit between known and predicted affinities.

	Split 1 Test	Split 2 Test	Split 3 Test	Mean ± STD
CEN	0.7008	0.6868	0.6876	0.6917 ± 0.0079
|*smina*|	0.2516	0.2995	0.3275	0.2929 ± 0.0384
gnina default	0.5674	0.5472	0.5555	0.5567 ± 0.0102
dense	0.5411	0.5221	0.5321	0.5318 ± 0.0096
gnina2018	0.4973	0.4873	0.4932	0.4926 ± 0.0050
crossdock_default2018	0.4816	0.4542	0.4617	0.4659 ± 0.0141
redock_default2018	0.4978	0.4741	0.4815	0.4845 ± 0.0121
default2017	0.4176	0.4258	0.4169	0.4201 ± 0.0049

The training data were randomly split into thirds; for each fold, we trained a CEN model on two thirds of the data and tested on the remaining third (top row). For comparison, we also rescored the test-set examples with *smina* and *gnina*-based scoring functions (subsequent rows). In the case of the *smina* scores, we considered the absolute values.

CENsible Performance on the CASF-2016 Benchmark

As stressed in the main text, CENsible is designed to provide *interpretable* insights into the contributions of specific physicochemical interactions that contribute to protein/ligand binding, given a correct ligand pose. Its primary goal is not to predict overall binding affinity but to evaluate the extent to which specific atomic interactions contribute to that final affinity prediction, suggesting new strategies for lead optimization. That said, we certainly acknowledge that CENsible must be reasonably effective at predicting affinity if we are to have any confidence in its interpretations.

We used the CASF-2016 benchmark core dataset to further assess CENsible accuracy and compare it to other scoring functions. This set contains 285 protein-ligand complexes taken from the PDBbind2016 dataset, in addition to decoy molecules selected for docking and screening evaluation[13]. We rescored the full core set using the *allcen3* CENsible model, the most updated version of the trained model available in the published GitHub repository.

CENsible ostensibly outperforms all other methods included in the CASF-2016 benchmark dataset in the scoring- and ranking-power benchmarks (Table S2). Given the "correct" (crystallographic) poses of known ligands, the scoring and ranking-power benchmarks assess how well a scoring function predicts experimental binding affinities and affinity rankings, respectively. These benchmarks thus closely match the task CENsible was trained to perform (i.e., assessing known/correct ligand poses to characterize the physicochemical properties and interactions that contribute to binding). That said, we note that the PDBbind2016 core set overlaps with CENsible's training set, so CENsible's performance on this benchmark is likely inflated. In contrast, ΔvinaRF[14], ostensibly the next-best scoring function (and the only other machine-learning-based model), was trained on a dataset that specifically excluded PDBs present in the CASF-2007 or CASF-2013 benchmarks.

For the sake of time, we did not retrain CENsible on a new dataset that excluded the PDBbind2016 core-set structures. However, the cross-validation studies described in the previous section give a sense of how much CENsible's performance on the scoring-power benchmark might be inflated. When trained on two-thirds of the PDBbind data and evaluated on the remaining (withheld) third, CENsible achieved Pearson correlation coefficients of ~0.7 (Table S1). This value is lower than the 0.893 measured on the CASF-2016 benchmark. Nonetheless, if a retrained version of CENsible tailored specifically to CASF-2016 had similar performance, it would still rank among the best scoring functions evaluated per the scoring-power metric.

In contrast, CENsible's performance on the docking-power benchmark was poor (Table S3). This benchmark assesses how well a scoring function can identify a true ligand's correct pose from among many computer-generated incorrect poses of the same ligand. The docking-power task differs meaningfully from the one CENsible was trained to perform. CENsible provides interpretable insights given a correct ligand pose. It was trained only on correct (crystallographic) poses, not incorrect poses.

Finally, CENsible's performance on the screening-power benchmark was also poor (Table S3). This benchmark assesses how well a scoring function can identify true ligands from a set that

includes many decoy molecules (presumed non-binders). This task also differs meaningfully from the one CENsible was trained to perform. Specifically, CENsible was trained on known-ligand poses and is not necessarily suited to the tasks of assessing incorrect poses or the poses of compounds that do not bind to the target.

Table S2. Comparison of performance between CENsible and all scoring functions included in CASF- 2016 benchmark.

Scoring function	Scoring power (Pearson R)	Ranking power (Spearman R)	Ranking power (Predictive index)
**CENsible**	**0.893**	**0.802**	**0.826**
ΔVinaRF20	0.816	0.750	0.761
X-Score	0.631	0.604	0.638
X-ScoreHS	0.629	0.547	0.577
Δ SAS	0.625	0.588	0.612
X-ScoreHP	0.621	0.573	0.607
ASP@GOLD	0.617	0.553	0.582
ChemPLP@GOLD	0.614	0.633	0.657
X-ScoreHM	0.609	0.603	0.641
AutodockVina	0.604	0.528	0.557
DrugScore2018	0.602	0.607	0.637
DrugScoreCSD	0.596	0.63	0.663
ASE@MOE	0.591	0.439	0.466
ChemScore@SYBYL	0.590	0.593	0.617
PLP1@DS	0.581	0.582	0.605
ChemScore@GOLD	0.574	0.526	0.558
G-Score@SYBYL	0.572	0.591	0.609
Alpha-HB@MOE	0.569	0.535	0.558
PLP2@DS	0.563	0.589	0.617
Affinity-dG@MOE	0.552	0.604	0.619
LigScore2@DS	0.540	0.608	0.620
D-Score@SYBYL	0.531	0.577	0.598
LUDI2@DS	0.526	0.629	0.657
GlideScore-SP	0.513	0.419	0.425
LUDI3@DS	0.502	0.532	0.564
GBVI/WSA-dG@MOE	0.496	0.489	0.504
LUDI1@DS	0.494	0.612	0.640
GlideScore-XP	0.467	0.257	0.255
Jain@DS	0.457	0.521	0.545
LigScore1@DS	0.425	0.599	0.606
PMF@DS	0.422	0.537	0.559
GoldScore@GOLD	0.416	0.284	0.283
London-dG@MOE	0.405	0.593	0.609
PMF@SYBYL	0.262	0.449	0.478
PMF04@DS	0.212	0.481	0.497

CENsible ostensibly outperforms all other scoring functions in scoring and ranking power. All benchmarks were performed using the crystallographic poses. Scoring power is measured as the Pearson correlation between computed scores and experimentally confirmed binding affinities. Ranking power is measured as the Spearman's rank correlation coefficient or the Predictive Index (see ref. 13).

Table S3. Additional performance comparison between CENsible and all scoring functions included in the CASF-2016 benchmark.

Scoring function	Docking power (Top 1 / Top 2 / Top 3)	Screening power (Top 1% / Top 5% / Top 10%)
**CENsible**	**23.9% / 37.2% / 44.6%**	**0.64 / 1.09 / 1.27**
ΔVinaRF20	89.1% / 94.4% / 96.5%	11.73 / 4.43 / 3.10
X-Score	63.5% / 74.0% / 80.4%	2.68 / 1.31 / 1.23
X-ScoreHS	59.6% / 72.3% / 78.6%	2.17 / 1.26 / 1.26
ΔSAS	30.2% / 44.6% / 51.6%	1.76 / 1.12 / 1.15
X-ScoreHP	56.1% / 67.7% / 75.1%	1.79 / 1.54 / 1.13
ASP@GOLD	81.1% / 88.4% / 93.0%	6.98 / 3.95 / 3.10
ChemPLP@GOLD	86.0% / 93.7% / 96.1%	11.91 / 5.29 / 3.59
X-ScoreHM	65.3% / 77.9% / 83.5%	3.21 / 1.39 / 1.31
AutodockVina	90.2% / 95.8% / 97.2%	7.70 / 4.01 / 2.87
DrugScore2018	83.5% / 89.5% / 94.0%	3.66 / 2.25 / 1.89
DrugScoreCSD	87.4% / 93.3% / 95.1%	5.90 / 2.97 / 2.54
ASE@MOE	50.5% / 60.7% / 67.0%	1.44 / 1.11 / 1.28
ChemScore@SYBYL	57.9% / 68.8% / 77.2%	0.79 / 1.26 / 1.41
PLP1@DS	82.8% / 90.5% / 94.0%	3.98 / 2.88 / 2.39
ChemScore@GOLD	80.4% / 86.0% / 90.9%	8.65 / 3.95 / 2.92
G-Score@SYBYL	44.2% / 59.6% / 69.1%	0.89 / 1.06 / 1.11
Alpha-HB@MOE	71.6% / 81.1% / 85.3%	1.70 / 1.65 / 2.02
PLP2@DS	79.3% / 88.4% / 92.3%	1.81 / 2.43 / 2.49
Affinity-dG@MOE	63.5% / 76.1% / 83.9%	5.07 / 2.77 / 2.26
LigScore2@DS	85.6% / 93.3% / 96.5%	6.82 / 3.53 / 2.84
D-Score@SYBYL	26.0% / 40.0% / 51.9%	1.24 / 1.65 / 1.34
LUDI2@DS	63.5% / 75.1% / 80.4%	2.34 / 2.00 / 1.63
GlideScore-SP	87.7% / 91.9% / 93.7%	11.44 / 5.83 / 3.98
LUDI3@DS	53.0% / 63.2% / 71.9%	1.85 / 1.15 / 1.40
GBVI/WSA-dG@MOE	87.0% / 91.9% / 93.3%	7.62 / 3.61 / 2.77
LUDI1@DS	63.2% / 73.7% / 81.1%	3.10 / 2.14 / 1.81
GlideScore-XP	83.9% / 90.2% / 94.4%	8.83 / 4.75 / 3.51
Jain@DS	55.8% / 67.4% / 75.8%	1.35 / 1.31 / 1.56
LigScore1@DS	76.8% / 86.0% / 89.5%	6.32 / 3.68 / 2.74
PMF@DS	42.8% / 51.9% / 58.6%	3.76 / 1.76 / 1.59
GoldScore@GOLD	75.1% / 86.3% / 90.5%	4.27 / 2.86 / 1.98
London-dG@MOE	63.2% / 78.2% / 83.9%	2.05 / 2.30 / 2.09
PMF@SYBYL	47.7% / 57.9% / 65.3%	1.46 / 1.77 / 1.68
PMF04@DS	46.3% / 54.0% / 59.6%	3.17 / 1.68 / 1.75

Docking power is measured as the success rate (percentage) of correctly ranked top-1, top-2, and top-3 near-native poses, with a near-native threshold of 2 Å. Screening power is measured as the enrichment of known actives in the top 1%, 5%, and 10% of compounds ranked by the respective scoring functions.

Similar CENsible Weight Vectors are Generally Adjacent in t-SNE Space

The t-SNE analysis presented in the main text is predicated on the assumption that similar predicted weight vectors, w_e, continue to be adjacent when projected into t-SNE space. To verify this assumption, we first used K-means clustering to identify groups of predicted weight vectors that are similar. We separately divided the data into 2, 3, 4, and 5 clusters and calculated silhouette coefficients for each clustering. These coefficients indicate the relative distance between a given example and its nearest neighbor not in the same cluster, such that high silhouette values suggest better cluster separation. We found that the best separation occurred when we clustered the data into two or three clusters (Figure S8).

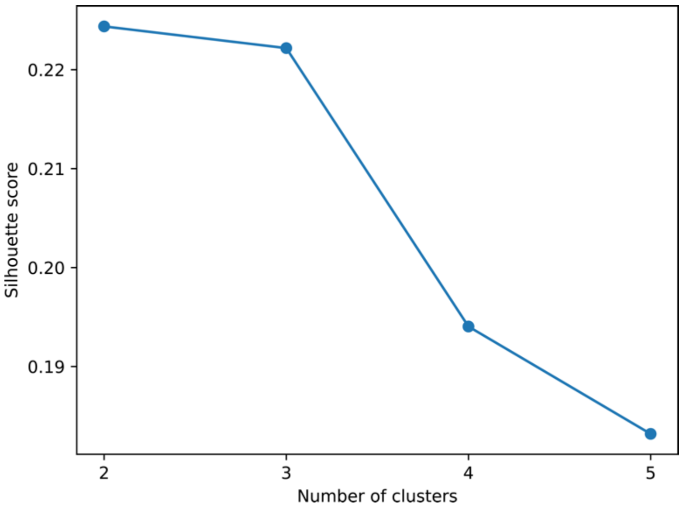

Figure S8. Averaged silhouette coefficient values when generating 2, 3, 4, and 5 clusters.

We next projected the members of these clusters into 2D t-SNE space to determine whether predicted weights belonging to the same cluster continued to be adjacent in the t-SNE space. Adjacency was largely preserved, especially when using two or three clusters (Figure S9, upper row), but also when dividing the data into 4 and 5 clusters (Figure S9, remaining panels). The analysis confirms that clusters are spatially correlated in the lower-dimensional space.

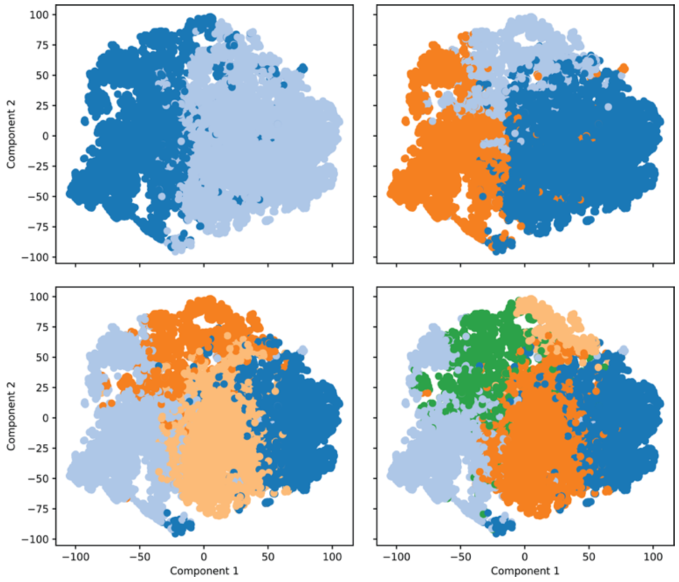

Figure S9. Predicted term weights projected onto 2D t-SNE space, colored by K-means cluster. Colors are randomly assigned.

References

1 Virtanen, P. *et al.* SciPy 1.0: fundamental algorithms for scientific computing in Python. *Nat Methods* **17**, 261-272 (2020). https://doi.org:10.1038/s41592-019-0686-2

2 Mann, H. B. & Whitney, D. R. On a test of whether one of two random variables is stochastically larger than the other. *The annals of mathematical statistics*, 50-60 (1947).

3 Shapiro, S. S. & Wilk, M. B. An analysis of variance test for normality (complete samples). *Biometrika* **52**, 591-611 (1965).

4 Student. The probable error of a mean. *Biometrika* **6**, 1-25 (1908).

5 Li, Y., Han, L., Liu, Z. & Wang, R. Comparative assessment of scoring functions on an updated benchmark: 2. Evaluation methods and general results. *J Chem Inf Model* **54**, 1717-1736 (2014). https://doi.org:10.1021/ci500081m

6 Cheng, T., Li, X., Li, Y., Liu, Z. & Wang, R. Comparative assessment of scoring functions on a diverse test set. *J Chem Inf Model* **49**, 1079-1093 (2009). https://doi.org:10.1021/ci9000053

7 Li, X., Li, Y., Cheng, T., Liu, Z. & Wang, R. Evaluation of the performance of four molecular docking programs on a diverse set of protein-ligand complexes. *J Comput Chem* **31**, 2109-2125 (2010). https://doi.org:10.1002/jcc.21498

8 Wang, Z. *et al.* Comprehensive evaluation of ten docking programs on a diverse set of protein-ligand complexes: the prediction accuracy of sampling power and scoring power. *Phys Chem Chem Phys* **18**, 12964-12975 (2016). https://doi.org:10.1039/c6cp01555g

9 Durrant, J. D. & McCammon, J. A. NNScore 2.0: a neural-network receptor-ligand scoring function. *J Chem Inf Model* **51**, 2897-2903 (2011). https://doi.org:10.1021/ci2003889

10 McNutt, A. T. *et al.* GNINA 1.0: molecular docking with deep learning. *J Cheminform* **13**, 43 (2021). https://doi.org:10.1186/s13321-021-00522-2

11 Sunseri, J. & Koes, D. R. Virtual Screening with Gnina 1.0. *Molecules* **26** (2021). https://doi.org:10.3390/molecules26237369

12 Koes, D. R., Baumgartner, M. P. & Camacho, C. J. Lessons learned in empirical scoring with smina from the CSAR 2011 benchmarking exercise. *J Chem Inf Model* **53**, 1893-1904 (2013). https://doi.org:10.1021/ci300604z

13 Su, M. *et al.* Comparative Assessment of Scoring Functions: The CASF-2016 Update. *J Chem Inf Model* **59**, 895-913 (2019). https://doi.org:10.1021/acs.jcim.8b00545

14 Wang, C. & Zhang, Y. Improving scoring-docking-screening powers of protein-ligand scoring functions using random forest. *J Comput Chem* **38**, 169-177 (2017). https://doi.org:10.1002/jcc.24667
