# Supplementary material for: CENsible: Interpretable Insights into Small-Molecule Binding with Context Explanation Networks
Source: J Chem Inf Model. 2024 Jun 7;64(12):4651–60. doi: 10.1021/acs.jcim.4c00825 (PMC11200255; doi:10.1021/acs.jcim.4c00825)
Supplement: Supplementary file 2 [file ci4c00825_si_003.pdf]

Grid Atom Types

Receptor

- AliphaticCarbonXSHydrophobe
- AliphaticCarbonXSNonHydrophobe
- AromaticCarbonXSHydrophobe
- AromaticCarbonXSNonHydrophobe
- Bromine_Iodine_Chlorine_Fluorine
- Nitrogen_NitrogenXSAcceptor
- NitrogenXSDonor_NitrogenXSDonorAcceptor
- Oxygen_OxygenXSAcceptor
- OxygenXSDonorAcceptor_OxygenXSDonor
- Sulfur_SulfurAcceptor
- Phosphorus
- Calcium
- Zinc
- GenericMetal_Boron_Manganese_Magnesium_Iron

Ligand

- AliphaticCarbonXSHydrophobe
- AliphaticCarbonXSNonHydrophobe
- AromaticCarbonXSHydrophobe
- AromaticCarbonXSNonHydrophobe
- Bromine_Iodine
- Chlorine
- Fluorine
- Nitrogen_NitrogenXSAcceptor
- NitrogenXSDonor_NitrogenXSDonorAcceptor
- Oxygen_OxygenXSAcceptor
- OxygenXSDonorAcceptor_OxygenXSDonor
- Sulfur_SulfurAcceptor
- Phosphorus
- GenericMetal_Boron_Manganese_Magnesium_Zinc_Calcium_Iron
